# Evaluate five different diagnostic tests for dry mouth assessment in geriatric residents in long-term institutions in Taiwan

**DOI:** 10.1186/s12903-019-0797-2

**Published:** 2019-06-13

**Authors:** Yao-Ming Cheng, Shao-Huan Lan, Yen-Ping Hsieh, Shou-Jen Lan, Shang-Wei Hsu

**Affiliations:** 1 Department of Healthcare Administration, Asia University, Taichung City, Taiwan; 2 School of Pharmaceutical Sciences and Medical Technology, Putian University, Putian City, China; 3 Department of Long-term Care, National Quemoy University, No. 1 University Rd., Jinning Township, Kinmen County 89250 Taiwan; 4 Department of Medical Research, China Medical University Hospital, China Medical University, Taichung, Taiwan

**Keywords:** Dry mouth, Long-term care institution n, Oral moisture, Decision tree

## Abstract

**Background:**

Residents in long-term care (LTC) institutions require care plans to effectively resolve dry mouth. Simple and easily comprehensible dry mouth indices must be developed to assist care professionals in determining dry mouth among residents. Therefore, this study aim of the study was to evaluate five different diagnostic tests for dry mouth assessment.

**Methods:**

A total of 568 residents were recruited from several LTC institutions in central Taiwan. The research instruments and tools comprised of the characteristics of the residents, state of oral health care, self-perceived ability to chew food, Taiwanese short-form of the Oral Health Impact Profile (OHIP-7 T), self-perceived levels of dry mouth, oral moisture checking, and a repetitive saliva swallowing test (RSST). The data collected were analyzed through demographic analysis, Correlation coefficient and chi-squared automatic interaction detection.

**Results:**

Results of the decision tree analysis indicated that RSST results, tooth brushing frequency, and age were the three indices that exerted the greatest influence on oral moisture levels. Specifically, in residents with relatively high RSST results, a daily tooth brushing frequency > 1, and an age < 68 years exhibited more favorable oral moisture levels. The results indicated that residents’ self-perceived oral status was not associated with their oral moisture levels.

**Conclusion:**

The three indices can be provided to LTC institutions for on-site assessment of dry mouth among residents to facilitate early detection of those with dry mouth.

## Background

Residents of long-term care (LTC) institutions usually have poor oral health [1], with dry mouth being one of the primary oral diseases. In addition to the effect of diseases, 20 to 86% of cases of dry mouth co-occur with salivary hypofunction status [2–4]. The symptomatic dry mouth that can have multivariate negative effects, including chewing difficulty, dysphagia, loss of appetite, malnutrition, oral discomfort, risk of tooth decay, burning-mouth sensation, halitosis, less social interaction, and lowered quality of life [5–11]. Numerous factors influence the occurrence of dry mouth in institutional elderly, and Japanese studies have reported factors such as body mass index (BMI), physical disability, age, mouth breathing, frequency of daily brushing, sex, medication, tube feeding, and level of conversation [12–14].

In addition, an Australian study proposed mechanisms for the management of dry mouth among LTC residents [15], and a Brazilian study reported that residents with dysphagia had dry mouth symptoms greater in number [16].

Studies have suggested that older adults who received nonpharmacological interventions, such as participation in an oral function training program (e.g., massage on salivary gland and face, and tongue muscle training), showed improvement in dry mouth and overall oral status [2, 17, 18]. This demonstrated that older adults’ regular participation in oral function training programs is a crucial preventative measure that ensures good oral health. However, improving the ability of caregivers in long-term care institutions to evaluate dry mouth symptoms in residents is a prerequisite for such programs. If caregivers can evaluate dry mouth symptoms in institution residents, implementing comprehensive oral health care strategies is possible (including oral medical care and treatment, daily oral cleaning, and oral functional training).

However, implementing oral care in LTC institutions in Taiwan is a challenge. First, oral treatment by dentists is mainly based in hospitals or private clinics, and conducting oral care in LTC institutions is still in the stage of policy encouragement. This is mainly because of the lack of vehicles for transporting residents, inadequate space or medical equipment for installing dental chairs at LTC institutions, and medical insurance benefits [19–21]. Second, in terms of caring for the oral conditions of LTC residents, dental hygienists have the functions of educating, preventing oral diseases, and promoting oral health and hygiene [22]. For example, in countries such as Japan, South Korea, Sweden, the United States, Australia, and Brazil, dental hygienists provide oral care and examinations as well as improve or assist in the oral knowledge and skills development of institutional caregivers [23–26]. However, Taiwan does not have such a system for oral hygienists [27]. Therefore, the task of promoting the oral health of LTC residents is mainly conducted by institution staff. Factors such as the inadequate knowledge and experience of caregivers in oral care [28, 29] and oral care services are not top priorities [30, 31], which affects the willingness of staff to provide oral care to LTC residents.

Therefore, educating and training of knowledge and abilities regarding care for oral diseases among professionals is essential and thus helps improve the oral health of residents [32, 33]. However, such training is difficult to implement in LTC institutions that lack oral health education measures and oral care professionals. Therefore, the formulation of a simple and convenient dry mouth indicator is necessary. Such an indicator can assist care professionals in accurately evaluating dry mouth among residents. Chalmers et al.(2005) reported that attention to saliva status is a crucial training component for caregivers to develop their ability to evaluate oral health [34] and is an objective indicators of dry mouth [15].

This study aim of the study was to evaluate five different diagnostic tests for dry mouth assessment. These were evaluating the residents’ characteristics, self-perceived ability to chew food, oral health impact profiles, and self-perceived levels of dry mouth, as well as a repetitive saliva swallowing test.

## Methods

### Study design and participants

The study design is cross-sectional. We explained our research to 27 appropriated registered LTC institutions in central Taiwan, fifteen of which are recruited in this study intern recruited their residents as research participants. This study recruited reviewers who received training regarding the purpose of the study and content of the questionnaire as well as techniques to measure saliva, the required interview skills, and necessary precautions. These reviewers conducted face-to-face interviews with LTC residents and collected questionnaire data.

The inclusion criteria were residents who had (1) excluded those who are diagnosed as neurocognitive disorder and were willing to have written informed consent; (2) lived in LTC institutions in central Taiwan for at least 1 month and were aged ≥50 years, and (3) could provide clear responses. A total of 577 eligible residents were identified, 568 of them were willing to participate. Of the 568 copies of the questionnaire distributed, 560 valid responses were received.

The study was presented to the Institutional Review Board of the Cheng Ching General Hospital, and was approved (HP140026). The data were collected from August 2014 to March 2015.

### Measurements

The research instrument was a self-developed questionnaire created on the basis of previous studies and scales regarding dry mouth [7, 33, 35–37]. The content validity was evaluated by three experts with experience of performing oral care services in LTC institutions (two nurses in LTC institutions and one dentist). The reliability was assessed using the Cronbach’s α coefficient or the Kuder–Richardson formula 20.

The information obtained from the questionnaire survey comprised the residents’ demographic attributes, such as gender, educational attainment, age, type of LTC institutions lived in, marital status, average length of LTC stay (months), daily water intake (mL), number of diseases experienced in the last 6 months, and state of oral health care, (i.e., frequency of tooth brushing and gargling per day, and whether the residents had received teeth cleaning in the previous 6 months).

Questionnaire responses confirmed that the residents’ textures of food intake in the LTC institutions were divided into four categories: (1) a soft foods (for older adults with poor chewing ability but normal swallowing function); (2) a finely chopped foods (solid food is processed through methods such as mincing and grinding to produce food that can be swallowed without chewing); (3) a semi-liquid foods (solid food is processed through methods such as mincing and grinding, after which it is added to porridge, soup, and drink to that can be swallowed with or without a little chewing); and (4) a fully liquid foods (semiliquid food is completely liquidized in a juicer). The Barthel Index was used to verify LTC residents’ 10 activities of daily living (ADL); their scores ranged from 0 to 100, with a lower score indicating higher reliance on others’ assistance in daily life [38].

The residents’ body mass index (BMI) was calculated after their height in centimeter (cm) and weight in kilogram (kg) were measured. The height of bedridden residents or those unable to stand was measured using their knee height (cm) [39]. Weight measurements were performed using a weighing scale borrowed from the LTC institution. The approximate amount of water in milliliter (mL) drunk by the residents per day was measured using a 350 mL cup (excluding the amount of water in meals).

#### Taiwanese short-form of the Oral health impact profile (OHIP-7 T) [40]

The Oral Health Impact Profile (OHIP-7 T) was used to measure the residents’ oral health status by asking residents about the problems they had experienced in the past year regarding their mouths, teeth, or dentures. Specifically, this instrument comprised the following seven questions: “have you ever experienced problems related to your teeth or dentures?”; “have you ever been interrupted in meal because of problems related to your teeth or dentures?”; “have you ever experienced discomfort because of problems related to your teeth or dentures?”; “have you ever had difficulties of concentrating because of problems related to your teeth or dentures?”; “have you ever experienced difficulties in pronunciation because of problems related to your teeth or dentures?”; “have you ever encountered difficulties in daily life because of problems related to your teeth or dentures?” and “have your sense of taste deteriorated because of problems related to your teeth or dentures?”. The residents replied these questions on a 4-point Likert scale (0 = *never* and 4 = *often*). The total possible score was 28 points. A lower score indicate more favorable oral health status. The Cronbach’s α of this study was 0.95.

#### Self-perceived ability to chew food [41]

A scale for self-perceived ability to chew food was used to assess the residents’ self-evaluated chewing ability. This scale incorporated 24 items: eight fruits were used to test the self-perceived ability of chewing foods with hardness; four fresh foods and meats were used to test the self-perceived ability of chewing foods with toughness; eight cooked vegetables were used to test the self-perceived ability of chewing foods with fracturability; and four viscous foods were used to test the self-perceived ability of chewing foods with viscosity. Table 2 presents the detailed food names. The foods in each category were ranked according to their difficulty to chew [41]. Those listed at the top were the most difficult to chew, and those at the bottom were the easiest to chew. The residents were asked about the difficulty of chewing each category of food, and 1 points were assigned if their response was “easy to chew,” whereas 0 points were assigned if their response was “difficult or unable to chew.” The highest score for each category is 8 points, yielding a total score of 32 points (total scores of fresh foods, meats, and viscous foods multiplied by 2). A higher score suggested more satisfactory chewing ability. The value of the Kuder–Richardson formula 20 was 0.946, indicating moderate reliability.

#### Self-perceived levels of dry mouth

Another scale was conceived to measure residents’ self-perceived levels of dry mouth. A total of nine items were designed, which were evaluated using a 5-point Likert scale (1 = *never* and 5 = *frequently*). The total score is 45 points; a higher score indicated higher levels of self-perceived dry mouth. This scale examine residents self-perceived symptoms of dry mouth in the last month with the following statements: “I feel dryness in my mouth”; “I feel dryness in my lips”; “I feel that my gums are swollen, hurt, or have a burning sensation”; “I need fluids (e.g., soup or water) to help me swallow my food”; “I need to get up at night to drink water”; “I often feel like my mouth is dry after having a meal;” “I have difficulty eating food without water content”; “I feel dryness in my nose”; and “I feel dryness in my eyes.” The Cronbach’s α was 0.80.

#### Oral moisture checking

**Fig. 1:**
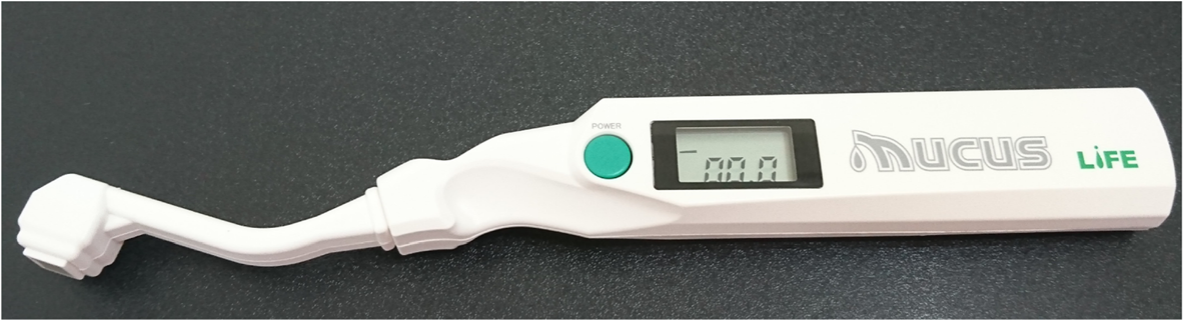
Oral moisture checking device (Moisture®, approval number: 22200BZX00640000, Life Co., Ltd., Saitama, Japan)

**Fig. 2:**
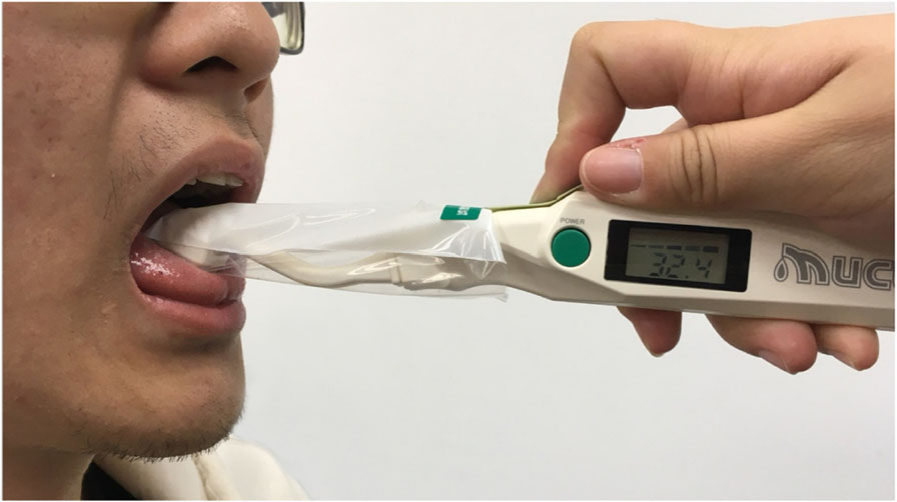
Measurement of oral moisture degree

Researches have indicated that decreased oral moisture levels correlate with increased oral dryness [42–44]. Therefore, we measured the actual moisture levels in the mouth of the residents to determine their levels of dry mouth. An oral moisture checking device (Moisture®, approval number: 22200BZX00640000, Life Co., Ltd., Saitama, Japan) [45] was used for the measurement. As shown in Fig. 1, the sensor on the left was used to detect the moisture of the oral cavity and the green button was for the measurement; the right side of the figure shows the data displayed after the measurement. Because this device is easy and quick in measurement, it is suitable for use among residents in LTC institutions [43, 46]. Measurement was conducted according to the following steps:

1. The surface of the tongue 10 mm from the apex linguae was measured. The measurement for each resident was conducted using a new sense cover. For each measurement attempt, approximately 200 g of pressure was applied using the device, and a value was then provided after 2 s. Three consecutive readings were taken, and the median of these is the final measurement. Figure 2 presents the measurement using the oral moisture checking device.
2. The residents were requested to sit and rest for 5 min before the measurement. With reference to the suggestion provided by Saito et al. (2008), the measurement was conducted between two meals, ideally from 10:00 AM to 11:00 AM or from 2:30 PM to 4:30 PM [47].
3. Definitions of the measurement values were as follows: ≥29.6 was defined as normal, ≤27.9 was defined as dry mouth, and 28.0–29.5 was defined as borderline dry mouth [43].

#### Repetitive saliva swallowing test (RSST)

The repetitive saliva swallowing test (RSST) was employed to determine the residents’ swallowing movements. Oguchi et al. (2000) suggested that RSST is a simple and non-invasive examination [48]. In this test, research personnel assessed the residents’ frequency of saliva swallowing by touching their prominentia laryngea through palpation and counting the frequency of swallowing over the span of 30 s. Residents who swallowed saliva three times or more within 30 s were considered to have normal swallowing ability. Those who swallowed saliva only one or two times were considered to have a moderate risk of dysphagia, and those who could not successfully swallow saliva or choked during the process were considered to have a high risk of dysphagia [49, 50].

### Statistical analyses

Statistical analysis was performed by using SPSS version 19. Descriptive statistics including frequencies, percentages, mean and 95% confidence interval (CI) were reported. The correlation coefficient was used to confirm the correlation level between the oral moisture measurement and other variables.

Chi-squared automatic interaction detection (CHAID) was employed to create a decision tree. Chi-square automatic interaction detection (CHAID) is a nonmathematical decision tree that apply the stepwise classification method (tree classification method) to detect interactions. A CHAID technique involves a tree analysis between one dependent variable and several independent variables. This method enables classifying both continuous and categorical variables. CHAID operates by examining dependent variables and independent variable through a chi-square test to identify significant variables. If no significant difference exists between the sequential groups of selected independent variables, these variables are combined as one group. That is, each interval of that variable is automatically separated as being mutually independent from other variables through category merging [51].

The dependent variable of this study was oral moisture (interval variable). The independent variable consisted of the residents’ demographic characteristics, oral health status, OHIP-7 T results, self-perceived ability to chew food, self-perceived level of dry mouth, and RSST results. We input all independent variables in a CHAID model and did not conduct a hierarchical analysis. To determine the selection sequence of independent variables, this study adopted the automatic model selection method of CHAID. SPSS was used to identify the most significantly lowest probability value in order to divide variables into groups. Likelihood ratio was used in the chi-squared tests. To prevent overfitting, the minimum parent node size was set at 100 and the minimum child node size was set as 50. The eligibility level and merge level were set to a *p*-value threshold of .05 [51, 52].

## Result

Table 1 presents the residents demographic information. Of all 560 sampled residents, the majority (64.6%) lived in nursing homes. Their mean ADL score was 67.71 (95% CI, 65.28–70.14), indicating that most of these residents had mild disabilities. Female residents constituted the majority (55.4%). The residents had an average age of 77.1 years (95% CI, 76.14 year - 77.94 years), low educational attainment (79.6%) were illiterate or had only received an elementary school education), and were experiencing multiple diseases (mean = 2.08; 95% CI: 1.96–2.20). Their average length of stay in LTC was approximately 4 years (mean = 45.7 months; 95% CI: 42.57 months - 48.99 months). The residents’ body mass index were within the normal range (mean = 22.91; 95% CI: 22.60–23.24). The residents’ average ADL score was 67.71 (95% CI, 65.28–70.14), indicating they required minor help performing ADLs. The texture of food intake at the institution was mainly soft (53.0%). Furthermore, their self-estimated quantity of daily water intake was approximately 899.15 mL on average (95% CI, 851.15 mL – 947.17 mL).

**Table 1:** The Characteristics of the residents

Variable	Number of residents (Percentage)	Correlation coefficient between oral moisture and each variable
Gender		
Male	250 (44.6)	−0.03
Female	310 (55.4)	
Education		
Illiterate	223 (39.8)	−0.07
Elementary school	220 (39.3)	
Junior high school	58 (10.4)	
High school	43 (7.7)	
College degree	16 (2.9)	
Type of LTC institutions		
LTC institutions	15 (2.7)	−0.02
Residential Care Home	362 (64.6)	
Nursing home	183 (32.7)	
Marital status		
No spouse	71 (12.7)	0.03
Marital status	131 (23.4)	
Divorced	51 (9.1)	
Widowed / separated	307 (54.8)	
Texture of food intake		
Soft foods^a^	297 (53.0)	
Finely chopped foods^b^	133 (23.8)	−0.09^*^
Semi-liquid diets^c^	96 (17.1)	
Full liquid diets^d^	34 (6.1)	
Variable	Means (95% CI)	Correlation coefficient between oral moisture and each variable
Average length of LTC stay (months)	45.78 (42.57–48.99)	0.02
Number of diseases experienced	2.08 (1.96–2.20)	0.06
Height (cm)	157.57 (156.80–158.34)	0.02
Body weight (kg)	56.95 (56.05–57.84)	0.06
Body Mass Index (BMI)	22.91 (22.60–23.24)	0.06
Daily water intake (mL)	899.15 (851.15–947.17)	0.03
Activities of daily living scale (ADL)	67.71 (65.28–70.14)	0.14^**^

Note: ^a^ soft foods: for older adults with poor chewing ability but normal swallowing function. ^b^finely chopped foods: solid food is processed through methods such as mincing and grinding to produce food that can be swallowed without chewing. ^c^ semi-liquid foods:solid food is processed through methods such as mincing and grinding, after which it is added to porridge, soup, and drink to that can be swallowed with or without a little chewing. ^d^ fully liquid foods: semiliquid food is completely liquidized in a juicer. cm: centimeter. *kg* kilograms, *BMI* Body mass index, *mL* milliliter, *ADL* Activities of daily living, *CI* confidence interval. * significant at *p* < 0.05; ** significant at *p* < 0.01. *N* = 560

All residents performed oral health care at least once per day in the form of brushing their teeth or gargling. However, 90% residents have not received dental examination within the past half year, suggesting that few of them visited dental clinics to examine their oral health status. In addition, The Self-perceived ability to chew food 的mean total score was 20.22 (95% CI, 19.45–20.98), indicating a moderate–high level of chewing ability (Table 2). The four categories of food each had a score of 8 points, and the residents’ self-perceived chewing ability had a score exceeding 4 points, indicating that they still possessed satisfactory chewing ability. The mean score of chewing foods with fracturability (cooked vegetables) was the most satisfactory (5.87, 95% CI, 5.66–6.07), whereas the mean score of chewing foods with toughness (fresh foods and meats) was the least satisfactory (4.40, 95% CI, 4.17–4.60).

**Table 2:** State of oral health care and Self-perceived ability to chew food

Variable	Number of residents (Percentage)	Means (95% CI)	Correlation coefficient between oral moisture and each variable
1. State of oral health care			
(1) Regular dental examination in the previous 6 months			
NO	511 (91.3)	–	0.06
Yes	49 (8.8)	–	
(2) Frequency of tooth brushing per day	–	1.65 (1.56–1.75)	0.11^*^
(3) Frequency of gargling per day	–	1.32 (1.20–1.44)	0.12^**^
2. Self-perceived ability to chew food		Means (95% CI)	Correlation coefficient between oral moisture and total score
*Fruits* (*non-pickled fruits, overripe/soft fruits, or juice*)			
1.Sugar cane (not juice)	–	5.24 (5.05–5.43)	–
2.Sliced guava	–		
3.Sliced apple or pear	–		
4.Sliced orange	–		
5.Sliced star fruit or bell fruit	–		
6.Sliced melon or tangerine	–		
7.Sliced watermelon or pineapple	–		
8.Papaya or banana	–		
*(2) Fresh foods and meats*			
1.Squid	–	4.40 (4.17–4.60)	–
2.Soy sauce-braised pork ears	–		
3.Fried chicken leg or chicken fillet	–		
4.Fish (steamed)	–		
*(3) Cooked vegetables* (*mainly scrambled or braised food*)			
1.Stir-fried peanut	–	5.87 (5.66–6.07)	–
2.Boiled bamboo shoots or broccoli	–		
3.Sliced cucumber or kidney bean	–		
4.Water spinach or cabbage	–		
5.Pickled lettuce in soy sauce or pickled cucumber in soy sauce			
6.Sliced sweet pepper	–		
7.Steamed sweet potato or taro	–		
8.Overcooked tomato	–		
*(4) Sticky foods*			
Malt syrup nougat	–	4.73 (4.45–5.00)	–
Mochi	–		
Rice cake	–		
Rice dumpling	–		
Total score	–	20.22 (19.45–20.98)	0.07

Note: *N* = 560. * significant at *p* < 0.05; ** significant at *p* < 0.01. CI: confidence interval

The average score of the residents’ OHIP-7 T was 3.76 (95% CI, 3.25–4.28), indicating that they possessed satisfactory oral health-related quality of life (Table 3). Moreover, the mean score for self-perceived level of dry mouth was 16.02 (95% CI, 15.57–16.50), indicating that on average they perceived dryness in their mouth (Table 4). The mean of oral moisture measurement was 27.97 (95% CI, 27.63–28.31), which also suggested mouth dryness. The mean frequency of saliva swallowing measured by RSST was 2.97, which was on the borderline between normal and dry mouth; indicating that overall, the residents may tend to experience some degree of dry mouth (Table 4).

**Table 3:** Taiwanese Short-form of the Oral Health Impact Profile

Items	Means (95% CI)	Correlation coefficient between oral moisture and total score
1. Have you ever experienced problems related to your teeth or dentures?	0.78 (0.68–0.87)	–
2. Have you ever been interrupted in the middle of a meal because of problems related to your teeth or dentures?	0.65 (0.56–0.74)	
3. Have you ever experienced discomfort because of problems related to your teeth or dentures?	0.64 (0.55–0.73)	
4. Have you ever had difficulties concentrating because of problems related to your teeth or dentures?	0.48 (0.40–0.56)	
5. Have you ever experienced difficulties in pronunciation because of problems related to your teeth or dentures?	0.45 (0.37–0.53)	
6. Have you ever encountered difficulties in daily life because of problems related to your teeth or dentures?	0.42 (0.34–0.49)	
7. Has your sense of taste deteriorated because of problems related to your teeth or dentures?	0.35 (0.28–0.42)	
Total score	3.76 (3.25–4.28)	−0.04

Note: *N* = 560. CI: confidence interval

**Table 4:** Self-perceived levels of dry mouth, Oral moisture checking and Repetitive saliva swallowing test

Items	Means (95% CI)	Correlation coefficient between oral moisture and total score
1. Self-perceived levels of dry mouth		
1.I feel dryness in my mouth	2.09 (2.00–2.18)	–
2.I feel dryness in my lips	2.00 (1.91–2.08)	
3.I feel that my gums are swollen, hurt, or have a burning sensation	1.31 (1.25–1.37)	
4.I need fluids (e.g., soup or water) to help me swallow my food	1.92 (1.82–2.01)	
5.I need to get up at night to drink water	2.07 (1.97–2.17)	
6.I often feel like my mouth is dry after having a meal	1.72 (1.65–1.80)	
7.I have difficulty eating food with no water content	1.93 (1.83–2.02)	
8.I feel dryness in my nose	1.36 (1.30–1.42)	
9.I feel dryness in my eyes	1.65 (1.58–1.73)	
Total score	16.02 (15.57–16.50)	−0.06
2. Oral moisture measurement	27.97 (27.63–28.31)	1
3. Repetitive saliva swallowing test	2.97 (2.84–3.11)	0.17^**^

Note: *N* = 560. ** significant at *p* < 0.01. CI: confidence interval

As shown in Tables 1, 2, 3 and 4, the relationships between oral moisture measurement and other variables were verified using the correlation coefficient. A total of five variables achieved significance, namely ADL (*r* = 0.14, *p* < 0.01), frequency of tooth brushing a day (*r* = 0.11, *p* < 0.05), frequency of gargling a day (*r* = 0.12, *p* < 0.01) and RSST (*r* = 0.17, *p* < 0.01); these were all positively correlated with oral moisture measurement. The texture of food intake (*r* = − 0.09, *p* < 0.05) were negatively correlated with oral moisture measurement. The correlation coefficient indicated a low level of correlation between oral measurement and other variables.

**Fig. 3:**
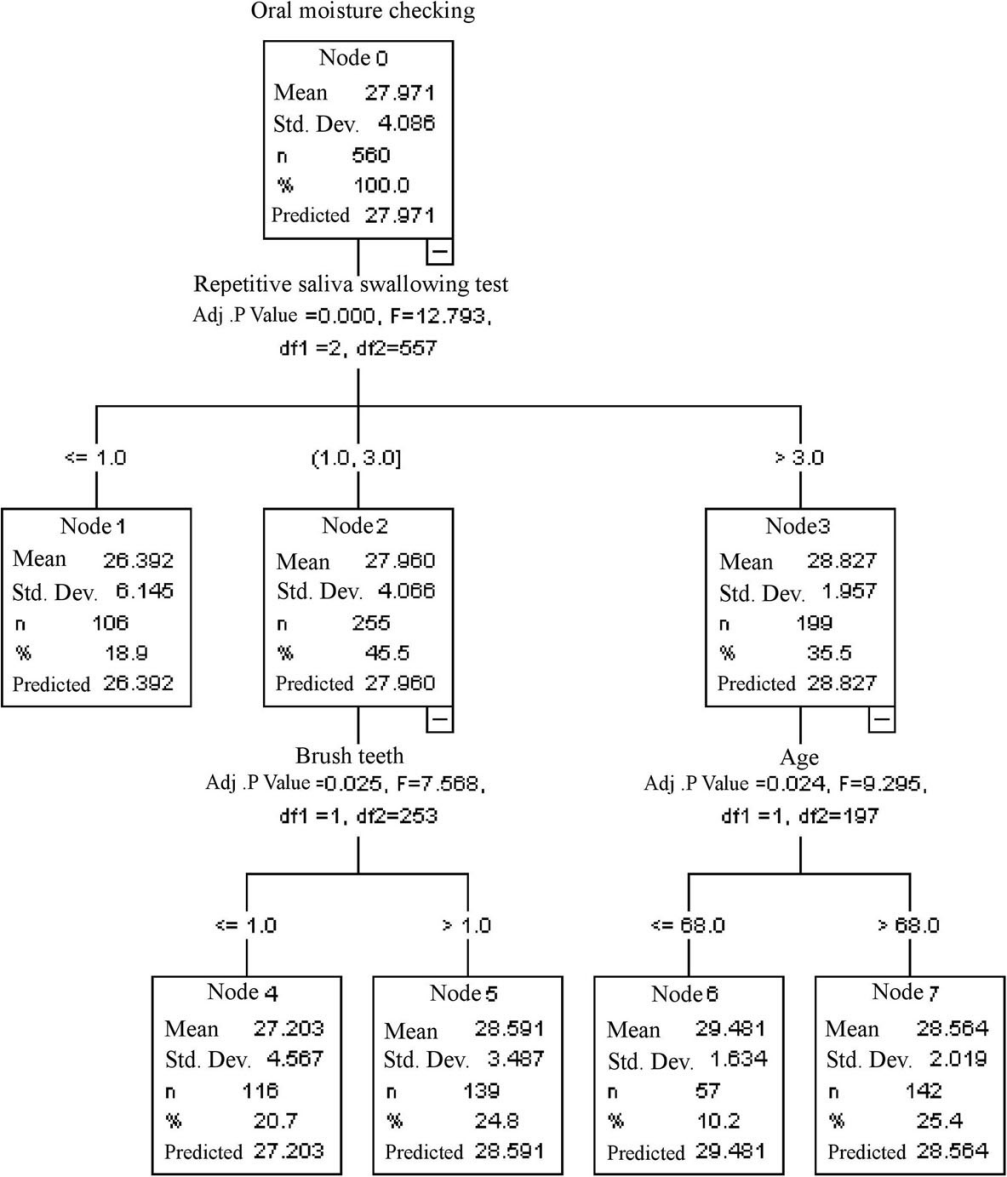
Decision Tree constructed by CHAID algorithm

Figure 3 shows the results of the CHAID decision tree analysis. Oral moisture measurement was the dependent variable and RSST results, tooth brushing behavior, and age were independent variables. The maximum tree depth was 3. The analysis revealed that RSST results was the most important variable (adjusted *p* < 0 .000; F = 12.793). RSST had three levels of risk: 1) A high risk of dry mouth suggested that the average value of oral moisture was SD: 26.39 and represented dry mouth level. The number of times saliva was swallowed was ≤1 (node 1): this indicated symptoms similar to that of dysphagia; 2) An average of oral moisture value of 27.96 indicated a moderate risk of dry mouth. The number of times saliva was swallowed was 1 to 3 (node 2); 3) An average oral moisture value of 28.827 suggested a low risk of dry mouth. This represented borderline dry mouth, and the number of times saliva was swallowed was over 3 (node3).

The number of times residents brushed their teeth was the next predictive variable for residents with a moderate risk of dry mouth (Adj. *p* < 0 .000, F = 7.568). Residents with a moderate risk of dry mouth who brushed their teeth more than once had an average oral moisture value of 28.59 (node5); this was higher than the average value of 27.20 (node 4) for residents who brushed their teeth once or less. This demonstrated that the number times residents brushed their teeth affected their oral moisture values if they had a moderate risk of experiencing dry mouth. For residents with a low risk of dry mouth, age is the next crucial predictive variable (adjusted *p* < 0.024, F = 9.295). The risk of experiencing borderline dry mouth in residents who were older than 68 years (the average oral moisture value was 28.564; node 7) was higher than that for residents who were aged 68 years or younger (the average oral moisture value was 29.481; node 6).

## Discussion

The researchers excluded residents who were confirmed to have neurocognitive disorders by physicians because of physical and psychological diseases, and explored the factors influencing the measurement of residents’ oral moisture levels. CHAID analysis revealed that the RSST result, tooth brushing frequency, and age were three best predictors for oral moisture levels. These finding can assist frontline care professionals in performing quick assessments of oral moisture levels among residents in LTC institutions.

The first significant predictor variable was RSST which correlated with the oral moisture levels of residents. Persson et al. (2018) used the absorbent method to collect residents’ saliva, and discovered that the measured saliva values did not affect the RSST [53], which was inconsistent with the results in the present study. The present authors believe this may be caused by different research methods producing different results. Dry mouth is one of the factors affecting oropharyngeal dysphagia [54]. Because dry mouth is a subjective feeling, people with sufficient and insufficient saliva secretion may report symptoms of dry mouth [55] and dysphagia [16]. The results of this study were similar to those of relevant studies in that higher oral moisture levels indicated more satisfactory swallowing function [16, 56].

Shunsuke et al. (2018) reported that a diagnosis of oral dryness was required when the oral moisture value (Mucus, Life Co., Ltd.) was < 27 [57]. This result is similar to that of node 1 in the present study; the average value of the residents’ oral moisture was lower than 26.392 and the RSST ≤1, indicating that node 1 residents experienced dry mouth and dysphagia. Therefore, caregivers should contact the oral medical team to conduct diagnosis and treatment of dry mouth among node 1 residents.

In addition, node 2 residents exhibited symptoms of dry mouth (average value of oral moisture = 27.96) and dysphagia (RSST = 1–3). The second significant predictor variable was tooth brushing frequency, which correlated with the oral moisture levels of residents of node 2. A brushing frequency of > 1 yielded an average oral moisture value of 28.591, which indicated borderline dry mouth; by contrast, a brushing frequency of ≤1 yielded an average oral moisture value of 27.203, which indicated dry mouth.

The authors believed that tooth brushing frequency was the protective factor for node 5 residents in maintaining oral moisture. Kakudate et al. (2014) reported that mechanical stimulation of the salivary glands during tooth brushing can increase the amount of saliva [58]. Studies have suggested that older adults whose brushing frequency was less than twice per day have a high probability of dry mouth [58, 59]; furthermore, switching to using an electric toothbrush increased the residents’ salivary flow [60], indicating that increased salivary flow helps smooth swallowing [56]. Additionally, the intervention results of oral function training programs indicated that they helped increase salivary flow and RSST [61, 62], suggesting that caregivers should focus more on oral hygiene and oral health promotion programs for node 2 residents.

Node 3 residents had borderline dry mouth (average oral moisture value = 28.827); however, they did not exhibit any symptoms of dysphagia (RSST > 3). The third significant predictor variable was age, which associated with the oral moisture levels of residents of node 3. Dry mouth is closely correlated with aging and salivary gland degeneration [3, 15]. Previous studies have used varying cut-off points for age. For example, some studies have identified older participants by age of ≥65 years, [11, 63] ≥75 years, [64] or ≥ 85 years [65, 66]. In the present study, age of 68 years was determined as the cut-off point. The results of this study might have been influenced by limited sample size or methodological differences. We found that the oral moisture level of residents with RSST results ≥3 and age ≤ 68 years were at approximately normal levels; however, those of residents age > 68 years were borderline. This finding furtherly verified that more attention should be directed to oral health status among residents aged > 68 years in LTC institutions even if they possess normal swallowing function.

We believe that the results highlight the importance of enhancing care professionals’ knowledge in LTC institutions that lack medical personnel specializing in oral health care or facilities for dry mouth assessment. Teaching such professionals about oral moisture checking and RSST methods, which help ensure the tooth brushing frequency and age indicators of residents, can assist caregivers in simply and conveniently providing assessment and oral care [49, 67]. In addition, collaboration with professional dental and medical teams can provide residents with more satisfactory oral health care.

This study had four research limitations. First, we excluded residents who were verified to have neurocognitive disorder by physicians, and the respondents’ average ADL score was 67.71 (95% CI, 65.28–70.14), indicating the residents’ need for minor help in performing ADLs. Therefore, the results cannot be extended to all LTC residents. However, because oral moisture checking instruments enable easy and quick assessment of residents’ level of oral dryness, such instruments can be used in the future to evaluate the level of oral dryness of patients who are bedridden, have severe disabilities, or have neurocognitive disorders.

Second, the aim of this study was to conduct a straightforward measurement of saliva moisture to examine factors influencing residents’ dry mouth status. Furthermore, the aim was to help institution personnel focus on simple observation indicators and provide oral health care assessments for residents. We did not specifically focus on the effect of different diseases on dry mouth because the level of dry mouth caused by systematic diseases (e.g., Sjögren syndrome, diabetes, kidney disease, and thyroid diseases) requires various differential diagnoses to be performed (e.g., serum biochemistry, examination of immunology, radiological examination of salivary glands, pathological examination, and examination of salivary cytokines) [68]. Furthermore, diagnoses and medical treatments must be performed by a professional medical team [69].

Third, most residents knew the functions of the medicine they should take, but were unable to correctly provide its product or scientific name. Therefore, this study did not account for the type and dosage of medications that the residents took. More than 500 types of medication can cause dry mouth, clinically such as antihistamines (e.g., diphenhydramine, and chlorpheniramine), anticholinergic agents (e.g., dicyclomine and oxybutynin), and hypotensive agents (e.g., captopril and methyldopa) [70]. Tan et al. (2018) reported that using medication with antimuscarinic properties for urinary frequency and incontinence and medication usage for the genitourinary and nervous systems in addition to antidepressants and psycholeptics were associated with dry mouth occurrence [71]. Moreover, the use of antihypertensive and cardiovascular drugs increases the risk of dry mouth [72]. A total of 70% of older adults in care institutions used more than 5 types of medications every day, which showed that the numbers and doses of medications are complex factors in such an analysis [73]. Future research should include doctor and pharmacy teams to evaluate the correlation between medication usage in older adults in care institutions and their dry mouth statuses.

Fourth, the self-developed questionnaires included “The Characteristics of the Residents,” “Self-perceived levels of dry mouth,” and “Self-perceived ability to chew food.” The questionnaire content may have featured a risk of bias and was not subject to verification by different groups. Although we adopted measures such as expert validity and statistical verification to ensure validity and reliability, some potential factors of dry mouth-related care were not considered. Therefore, the results may be biased and should be treated with caution.

Despite these limitations, this study provided empirical evidence for determining factors that influence levels of dry mouth among residents living in LTC institutions. The results can facilitate assessment of dry mouth among high-risk residents, thereby promoting individualized oral care measures. Our results provide frontline care professionals with a set of simple indices for dry mouth. Although the results indicated that the residents’ self-perceived oral statuses (i.e., oral health status, dry mouth status, and chewing ability) do not associated with their oral moisture levels.

Future research should explore other possible methods that could be employed for self-assessed detection of dry mouth. Moreover, the interaction effect between diseases of different levels and oral moisture is a pertinent topic for future studies.

## Conclusion

The research results verified that RSST results, tooth brushing frequency, and age were the three primary factors influencing dry mouth in the residents living in LTC institutions. RSST < 1 will result in symptoms of dry mouth and dysphagia. For residents who had borderline oral moisture levels and suspected swallowing disorder, dry mouth correlated with tooth brushing frequency. For residents aged > 68 years, oral moisture levels may be inadequate even with normal RSST results. Management and care professionals in LTC institutions should incorporate these finding into existing oral care services and evaluate the results.

## Data Availability

The datasets analyzed in this study are not publicly available because of older people confidentiality issues but are available from the corresponding author on reasonable request.

## Abbreviations

ADL: Activities of daily living
BMI: Body mass index
CHAID: Chi-squared automatic interaction detection
CI: Confidence interval
cm: centimeter
kg: kilograms
LTC: Long-term care
mL: milliliter
OHIP-7 T: Taiwanese short-form of the Oral Health Impact Profile
RSST: Repetitive saliva swallowing test
